# Risk of major depressive disorder in adolescent and young adult cancer patients in Japan

**DOI:** 10.1002/pon.5881

**Published:** 2022-01-20

**Authors:** Tatsuo Akechi, Izumi Mishiro, Shinji Fujimoto

**Affiliations:** ^1^ Department of Psychiatry and Cognitive‐Behavioral Medicine, Nagoya City University Graduate School of Medical Sciences Nagoya Japan; ^2^ Japan Medical Office, Takeda Pharmaceutical Company Limited Tokyo Japan

**Keywords:** adolescent, adult, cancer, depressive disorder, major, mood disorders, neoplasms, oncology, psycho‐oncology, young adult

## Abstract

**Objective:**

To estimate the risk of major depressive disorder (MDD) in adolescent and young adult (AYA) patients with cancer in Japan and identify risk factors for MDD among these patients.

**Methods:**

This was a matched cohort study using a large claims database in Japan. Included patients were aged 15–39 years, newly diagnosed with cancer during 2012–2017 and assessable for a follow‐up period of 12 months. Kaplan–Meier estimates and Cox proportional hazards models were used to calculate hazard ratios (HR) and 95% confidence intervals (CI) for MDD in the AYA patients with cancer versus age‐, sex‐ and working status‐matched cancer‐free controls. A subgroups analysis of the AYA patients with cancer was performed to explore MDD risk factors.

**Results:**

A total of 3559 AYA patients with cancer and 35,590 matched controls were included in the analysis. Adolescent and young adult patients with cancer had a three‐fold higher risk for MDD compared with cancer‐free controls (HR, 3.12; 95% CI, 2.64–3.70). Among cancer categories with >100 patients, patients with multiple cancer categories, including those with metastatic cancer (HR, 6.73, 95% CI, 3.65–12.40) and leukemia (HR, 6.30; 95% CI, 3.75–10.58), had the greatest MDD risk versus matched controls. Patients who received inpatient chemotherapy as initial treatment had a higher risk for MDD than patients without chemotherapy (HR, 0.43; 95% CI, 0.30–0.62).

**Conclusions:**

Adolescent and young adult patients in Japan with cancer are at high risk for MDD. Particularly, those with multiple cancer categories, leukemia, and those who receive aggressive anticancer treatments should be monitored closely for symptoms of MDD.

## 1 BACKGROUND

Cancer is a serious and potentially life‐threatening illness that can have profound effects on mental health.^1^ ^,^ ^2^ Most patients diagnosed with cancer experience a normal albeit painful emotional reaction; however, some develop clinical depression. Cancer patients with depression have poor cancer treatment adherence, increased healthcare utilization (including hospitalizations), and elevated risks for mortality and suicide.^3^, ^4^, ^5^, ^6^

Literature reviews have reported that 5%–10% of adult patients with cancer (aged ≥18 years) have experienced a major depressive episode (a rate 2–3 times higher than in the general population).^7^, ^8^ Additionally, observational studies and reviews have reported prevalence rates of depressive symptoms of up to 55% among adult patients with cancer (up to four times higher than the general population).^1^ ^,^ ^8^, ^9^, ^10^, ^11^, ^12^, ^13^ A nationwide matched‐cohort study conducted in Sweden found that the risk of mental disorders peaked during the first week after cancer diagnosis and that depression had the highest cumulative incidence among the various studied mental disorders (including stress reaction or adjustment disorder, substance abuse, or anxiety, somatoform or conversion disorders).^14^ In a previous study, we reported major depressive disorder (MDD) risk among adult cancer patients aged 18–74 years was almost tripled (hazard ratio [HR], 2.96; 95% confidence interval [CI], 2.77–3.16) compared with cancer‐free matched controls during an observation period from 6 months before to 12 months after cancer diagnosis.^15^ MDD risk was highest in patients with multiple cancer categories, most of whom had metastatic cancer, pancreatic cancer, and brain cancer. In the study mentioned above, 10.6% of included patients were aged ≤39 years, who are considered adolescent and young adults (AYAs).^16^

Adolescent and young adults (individuals aged 15–39 years) have limited access to dedicated institutions for healthcare and research.^17^ Hence, the risk of cancer and adverse cancer outcomes may be underestimated in this age group, which may have contributed to a lack of improvement in cancer survival rates over the years. Adolescent and young adult patients with cancers are at higher risk for depression compared with older adults.^12^ Observational studies from Asia, Germany, and the USA have reported that 25%–39% of AYA patients with cancer experience depressive symptoms.^18^, ^19^, ^20^ Data from Canada showed that AYA survivors of cancer had increased outpatient mental health visits and a higher risk of a severe psychiatric episode compared with matched controls.^21^ Furthermore, AYA patients with cancer carry a disproportionate burden of distress and psychological symptoms compared with older patients with cancer.^12^ A survey of 575 AYA patients with cancer revealed that although 90% of patients reported at least one emotional concern (66% reported depression or loss of interest in daily activities), only 43% of those with concerns sought help.^22^ Barriers to help‐seeking included not wanting to ask, embarrassment, and being told that their emotions were “normal.” Among those who sought help, 37% had difficulties obtaining assistance.

Despite previous findings, more robust data on the real burden of MDD among AYA patient with cancers, especially in comparison with cancer‐free controls and using large sample sizes, remains limited.^23^ A 2020 systematic review and meta‐analysis of psychiatric disorders in AYA cancer survivors could include only four and three studies, respectively, from 7934 studies screened.^16^ There are also significant knowledge gaps regarding the prevalence of MDD in AYA patients with different cancer categories and the various demographic and clinical variables associated with MDD risk in these patients. Such information would be essential in providing appropriate support for AYA patients with cancer. This study aimed to estimate MDD risk in AYA patients with cancer aged 15–39 years in Japan compared with matched cancer‐free controls.

## 2 METHODS

### 2.1 Study design and setting

This was a retrospective cohort study that included AYA patients with cancer in Japan, each matched to 10 cancer‐free controls. The ratio of 1:10 was in line with that used in a nationwide matched cohort study in Sweden.^14^ Data were retrieved from an administrative claims database housing multiple health insurance societies in Japan (JMDC database; JMDC Inc.).^24^ ^,^ ^25^ The JMDC database is an anonymized database on insured medical services and prescriptions. In Japan, all individuals are covered by either a national or an employer‐based comprehensive health insurance scheme.^26^ The JMDC database covers employees of medium to large companies and their non‐working family members aged <75 years. As a claims database, JMDC includes the definitive recorded diagnoses made by the patients' own healthcare providers and prescribed treatments.

The study protocol adhered to Ethical Guidelines for Medical and Health Research Involving Human Subjects in Japan^27^; because of the anonymized nature of the database and the retrospective nature of the study, institutional ethics approval and informed consent were not required.

### 2.2 Study population

Adolescent and young adult is defined as age 15–39 years, based on the 2006 Report of the AYA Oncology Progress Review Group of the US National Institutes of Health.^17^ The study included AYA patients with cancer in Japan with a new diagnosis of cancer from January 2012 to September 2017 (identification period) and cancer‐free controls matched by the month of cancer diagnosis (index), sex, age, and working status at index. A cancer patient was defined as having at least 2 records of the same cancer treatment within 3 months of a cancer diagnosis. The diagnosis of cancer was based on selected codes from the International Statistical Classification of Diseases and Related Health Problems, 10th revision (cancer: C00–C95), as listed in Table S1.^28^ Patients with diagnoses of multiple cancer categories were defined as those having ≥2 ICD‐10 cancer categories.

The study had an 18‐month observation period starting from 6 months before through 12 months after the month of cancer diagnosis (Figure S1). Exclusion criteria included any history of depression within 6 months before the start of the 18‐month observation period (window period), patient age <15 or ≥40 years, and having discontinuous health insurance enrollment (due to e.g., change of employer) before or after 12 months of the index months (study period).

Cancer‐free controls included those with no diagnosis of cancer during the study period and no history of depression during the window period.

### 2.3 Variables and assessment

For AYA patients with cancer, the following baseline characteristics were recorded: age, sex, working status (working vs. non‐working), category of cancer diagnosis, and initial treatment in the first 2 months of cancer diagnosis (chemotherapy [none, inpatient, or outpatient], radiation therapy [none, brachytherapy, or external beam radiotherapy], and presence or absence of surgery with ≥5 days' hospitalization). All AYA patients with cancer and matched controls were followed up for incident MDD, defined as either an ICD10 code of F32 (depressive episode) or F33 (MDD, recurrent) during the observation period.

### 2.4 Statistical analyses

Categorical variables were reported as counts and proportions whereas continuous variables were reported as means, standard deviations, medians, and ranges. A two‐sided significance level of 5% was used unless otherwise stated. Missing dates of prescriptions were imputed as the year and month of medical consultation. No other missing data were imputed.

Survival analysis was performed to estimate the time to the onset of MDD from the start of observation using the Kaplan–Meier method. The log‐rank test was used to compare survival curves. Cox proportional hazards regression analysis was performed to estimate the HRs and their 95% CI. Incidence of MDD in patients with cancer was compared against their matched cancer‐free control in (1) each cancer category (adjusting for sex and working status) and (2) all cancer categories combined (adjusting for sex, sex by age group [≥25 or <25 years], and working status). Furthermore, adjusted HRs for sex, sex by age group (≥25 or <25 years), working status, and initial treatment in the first 2 months of cancer diagnosis were calculated in all AYA patients with cancer (all categories combined).

## 3 RESULTS

A total of 3559 patients aged 15–39 years, with newly diagnosed cancer during the enrollment period and no recent history of depression were included in the AYA cancer group (Figure S2). The age‐matched cancer‐free control group included 35,590 individuals.

**FIGURE 1:**
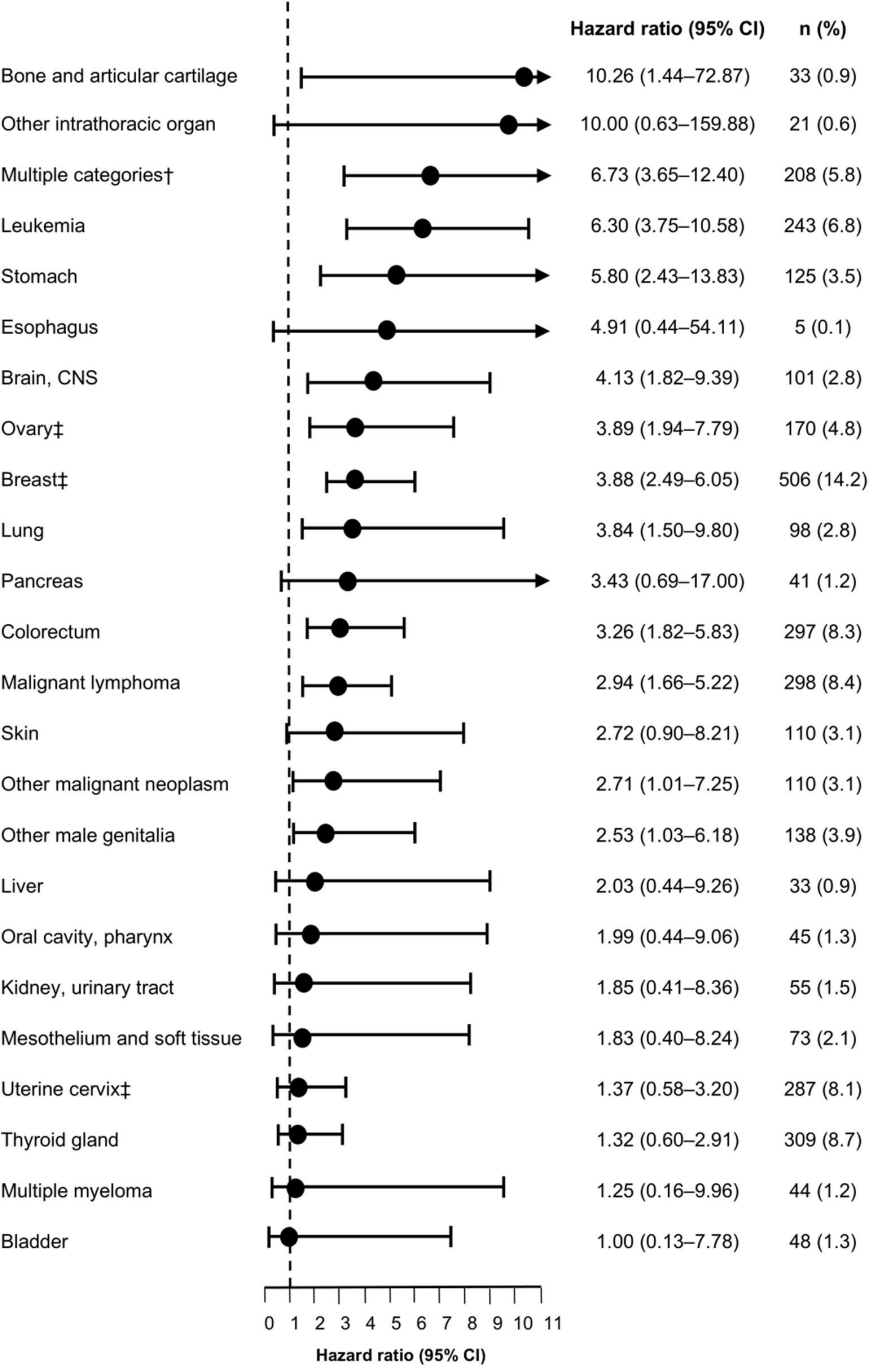
Risk of MDD in AYA patients with cancer versus their matched cancer‐free controls. HRs and 95% CIs of MDD were estimated by Cox proportional hazards model adjusting for sex and working status. The cancer‐free control populations were matched on age, sex, and working status. HRs for cancers of the gallbladder/bile duct, larynx, prostate, small intestines, uterine corpus, other digestive organs, nasal cavity, paranasal sinuses/middle ear, pharynx, and eye were not evaluable and are not shown here. †Defined as those diagnosed with ≥2 cancer categories by ICD‐10 codes; among patients with multiple cancer categories, those with “other malignant neoplasms (C76‐C80),” which includes metastatic cancer, were the most common. ‡Includes data from female patients only. AYA, adolescent and young adult; CI, confidence interval; CNS, central nervous system, HR, hazard ratio; MDD, major depressive disorder

There were more female than male AYA patients with cancer (59.9% vs. 40.1%) and more than half were full‐time workers (59.5%; Table 1). In both the cancer and control groups, 87% were aged ≥25 years. In the AYA patients with cancer, the most common cancers were breast cancer (14.2%; or 23.7% of all female patients), thyroid cancer (8.7%), malignant lymphoma (8.4%), colorectal cancer (8.3%), and leukemia (6.8%); 208 patients (5.8%) had diagnoses of multiple cancer categories (Figure 1). Of these patients, 194 had diagnosis of two cancer categories and 14 had ≥3. Among these patients, those with “other malignant neoplasms (C76‐C80),” which includes metastatic cancer, were the most common (145 patients with two sites and eight with ≥3 sites).

**TABLE 1:** Background characteristics of AYA patients with cancer and AYA controls

Variable, no. (%)	AYA patients with cancer (*n* = 3559)	AYA controls (*n* = 35,590)
Sex		
Male	1428 (40.1)	14,280 (40.1)
Female	2131 (59.9)	21,310 (59.9)
Age, years		
Mean (SD)	32.5 (6.3)	32.5 (6.3)
Median (range)	35 (15–39)	35 (15–39)
<25	464 (13.0)	4640 (13.0)
≥25	3095 (87.0)	30,950 (87.0)
Working status		
Working	2119 (59.5)	21,190 (59.5)
Non‐working	1440 (40.5)	14,400 (40.5)

*Note*: Data are presented as *n* (%), unless otherwise indicated.

Abbreviations: AYA, adolescent and young adult; SD, standard deviation.

**FIGURE 2:**
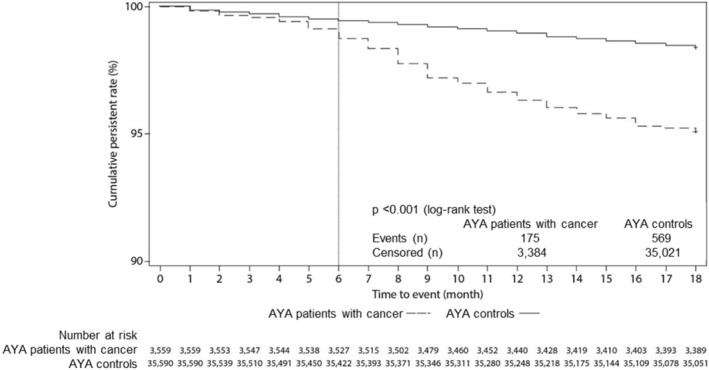
Kaplan–Meier analysis of time‐to‐MDD in AYA patients with cancer and their matched cancer‐free controls. AYA, adolescent and young adult; MDD, major depressive disorder

Among the AYA patients with cancer, 175 patients (4.9%) were diagnosed with MDD within the 18‐month observation period compared with 569 patients (1.5%) in the matched controls (log‐rank test *p* < 0.001). The time‐to‐MDD analysis for both groups is shown in Figure 2.

Multivariate analysis showed that AYA patients with cancer have an elevated MDD risk compared with cancer‐free patients (HR, 3.12; 95% CI, 2.64–3.70) after adjusting for covariates (Table 2). Furthermore, males aged ≥25 years had the highest risk of MDD (HR, 1.52; 95% CI, 1.05–2.21; with males aged <25 years as the reference) among the four sex/age categories (male vs. female/<25 vs. ≥25 years).

**TABLE 2:** Multivariate analysis of HRs of MDD incidence and 95% CI in AYA patients with cancer and matched controls

Variable	Reference	Category	Hazard ratio^a^ (95% CI)
Group	AYA control group	AYA patients with cancer	3.12 (2.64–3.70)
Sex	Male	Female	0.98 (0.83–1.15)
Sex, age (years)	Male, <25	Male, ≥25	1.52 (1.05–2.21)
		Female, <25	1.39 (0.90–2.16)
		Female, ≥25	1.35 (0.96–1.92)
Working status	Working	Non‐working	0.93 (0.77–1.11)

Abbreviations: AYA, adolescent and young adult; CI, confidence interval; HR, hazard ratio; MDD, major depressive disorder.

^a^ Adjusted for all covariates shown.

Cox proportional hazard regression analysis by cancer categories (with >100 patients) also showed that MDD risk was increased among patients with multiple cancer categories (HR, 6.73; 95% CI, 3.65–12.40); leukemia (HR, 6.30; 95% CI, 3.75–10.58), stomach cancer (HR, 5.80; 95% CI, 2.43–13.83); brain/central nervous system (CNS) cancer (HR, 4.13; 95% CI, 1.82–9.39); lung cancer (HR, 3.84; 95% CI, 1.50–9.80); colorectal cancer (HR, 3.26; 95% CI, 1.82–5.83); male genitalia cancer (HR, 2.53; 95% CI, 1.03–6.18), or malignant lymphoma (HR, 2.94; 95% CI, 1.66–5.22) compared with matched controls. Furthermore, female cancers were also associated with increased risk for MDD, as was observed with ovarian cancer (HR, 3.89; 95% CI, 1.94–7.79) and breast cancer (HR, 3.88; 95% CI, 2.49–6.05). The HRs for all analyzed cancer categories, including those with ≤100 patients, are reported in Table S2 and Figure 1.

Among the AYA patients with cancer, those who were not treated with chemotherapy had a lower MDD risk than those who had inpatient chemotherapy (HR, 0.43; 95% CI, 0.30–0.62; Table S3). Those who received external beam radiation therapy (HR, 1.35; 95% CI, 0.58–3.12) and those who underwent surgery and required ≥5 days of hospitalization (HR, 0.96; 95% CI, 0.70–1.33) did not have a significantly higher MDD risk.

## 4 DISCUSSION

This study shows that AYA cancer patients in Japan have a three‐fold higher risk of MDD within 6 months before and 12 months after cancer diagnosis compared with cancer‐free controls. To our knowledge, this is the first study to report MDD risk among AYA patients with cancer.

The results indicate an increased risk for MDD within 1 year of cancer diagnosis which aligns with findings from previous studies. The Behavioral Risk Factor Surveillance System (USA) also reported that among 4054 AYA cancer survivors (defined therein as aged 15–29 years upon cancer diagnosis), 20% reported poor mental health for ≥14 days in the 30 days before the survey compared with 10% among controls with no cancer history (*n* = 345,592).^29^ Data from the Danish Psychiatric Central Register of patients assessed from 1973 to 2003 (*n* = 121,227,396) found that the relative risk of depression among cancer patients ranged from 1.16 (95% CI, 0.90–1.51) in women with colorectal cancer to 3.08 (95% CI, 1.88–5.02) in men with brain cancer.^30^ However, it should be noted that although young patients (from age 15 years) were included in this registry, the upper age limit was undefined. Lastly, a Norwegian national cohort study found that among cancer patients aged 15–24 years, the risk of suicide was elevated compared with cancer‐free controls (HR, 2.6; 95% CI, 1.5–4.2).^31^ A heightened risk of MDD and suicide in these AYA patients versus cancer‐free controls, combined with the evidence that mental health issues are more likely in AYA than older cancer patients,^12^ suggests this population requires greater attention to prevent suicide, treat mental illness, and improve their quality of life.

The drivers of increased risk of MDD among AYA cancer patients remain unclear. However, some insights may come from research on post‐traumatic stress symptoms. One study noted that 44% of AYA cancer survivors experienced moderate‐to‐severe post‐traumatic stress symptoms.^32^ The researchers hypothesized that the need to cope with a life‐threatening disease aggravated the vulnerability that typically occurs during adolescence and young adulthood. Adolescent and young adult patients with cancer have reported concerns regarding sexuality, fertility, body image, and other psychosocial disruptions, which may contribute to the development of MDD.^12^ It is possible these same challenges play a role in the onset of MDD in AYA patients with cancer. Male patients aged ≥25 years were noted to have the highest MDD risk among the four age/sex categories of AYA (male vs. female/<25 vs. ≥25 years). Further study is needed to determine if this finding is specific to Japanese patients, as females are generally considered to have a higher MDD risk.

Analysis by cancer categories showed that the MDD risk in AYA patients with multiple cancer categories (HR, 6.73; 95% CI, 3.65–12.40) and brain/CNS cancer (HR, 4.13; 95% CI, 1.82–9.39) were significantly increased compared with cancer‐free controls. The same observation was noted among adult patients with cancer found in the JMDC database (HR, 5.89; 95% CI, 4.76–7.30 for multiple cancer categories, most of which were metastatic, and HR, 5.22; 95% CI, 3.13–8.71 for brain/CNS cancers).^15^ These results suggest that patients with these cancers should receive increased monitoring and mental health care.

In contrast, AYA patients with leukemia (HR, 6.30; 95% CI, 3.75–10.58) had a 1.6‐fold higher HR (vs. Adolescent and young adult controls) compared to that reported in our previous study among adult patients with leukemia versus adult controls (HR, 3.85; 95% CI, 2.74–5.40).^15^ Similarly, AYA patients with stomach cancer (HR, 5.80; 95% CI 2.43–13.83) had a 2.3‐fold higher HR (vs. Adolescent and young adult controls) than the HR reported in adult patients with stomach cancer versus adult controls (HR, 2.56; 95% CI, 2.02–3.24). These results may reflect more aggressive tumor behaviors, diagnosis at advanced stages, and the administration of more aggressive treatments for these cancers in AYA patients.^23^ ^,^ ^33^ Given the elevated MDD risk in these patients, special supportive measures should be considered.

Cancers with better prognoses (e.g., thyroid cancer) were generally associated with lower MDD risk, although patients with breast cancer, which has a 5‐year relative survival rate and conditional 5‐year relative survival rate of ≥90%,^34^ still had a four‐fold higher risk for MDD compared with matched controls. Several factors may contribute to this finding with breast cancer, including long‐term complications such as lymphedema, treatment‐related fatigue, and cognitive impairment, adverse effects of long‐term hormonal therapy, and sexual dysfunction.^35^ Similarly, patients with ovarian cancer experience unique psychological stresses, such as reduced sexual activity and satisfaction; fertility concerns; anxiety arising from the genetic association of the disease which could lead to fear for their female offspring; and the usual trajectory of ovarian cancer, which includes aggressive treatments and recurrence.^36^, ^37^, ^38^

Among the AYA patients with cancer, there was a significant 57% lower risk for MDD among those who did not receive chemotherapy as initial therapy compared with those who received inpatient chemotherapy. This finding mirrors the data in adult matched patients not receiving initial chemotherapy (HR, 0.53; 95% CI, 0.46–0.62).^15^ The increased risk of MDD among those who had inpatient chemotherapy may be related to the advanced stage of their disease.

Although the prevalence of mental disorders in Japan is lower than in Western countries,^39^ the matched cohort design of this study was able to quantify the burden in AYA patients with cancer compared with randomly selected matched controls. With the large sample size available, the study was able to sufficiently estimate the risk of MDD in AYA patients with cancer and examine the effects of age and sex on MDD risk. The availability of data from a large number of patients also allowed estimates of MDD risk across a broad range of cancer categories versus cancer‐free controls. Many of these cancers have not been examined previously.

### 4.1 Study limitations

To date, this study represents the most comprehensive assessment of the burden of MDD in AYA patients with cancer in Japan. However, it also has some limitations. In the claims database, diseases claimed without clinical definitions may lead to overestimation of their incidence. To increase the level of accuracy of cancer diagnosis, we selected those with ≥2 records of treatment within the first 3 months of diagnosis. For MDD, the level of misdiagnosis was not likely to differ between the cancer patients and the non‐cancer patient cohorts. Cancer patients who dropped out within 12 months of the index month were excluded, which may lead to underestimation of MDD risk in cancer patients.

Although the types of initial therapy (chemotherapy, radiotherapy, and surgery) were reported in the study, information on specific treatments by cancer categories was not analyzed in this study. The number of AYA patients with rare cancers was insufficient for inclusion in the analysis by cancer category. Furthermore, due to the nature of the database, we could not differentiate patients with metastatic/non‐metastatic disease. It was not possible to compare MDD severity as well as related factors such as prognosis or history of cancer or MDD. Previous publications have documented that rates of depression can be higher with certain types of cancer^40^ and that prognosis may also impact MDD rates.^41^ Further investigation of these points is warranted in future publications.

The risk of having had an MDD episode increases with age and the prevalence of MDD has also been reported to vary based on age.^42^ These limitations should be taken into consideration when interpreting the results. The age range selected for this study is in line with a previously published report investigating AYA patients with cancer,^16^ however, as it is broad, detecting differences in narrower patient age brackets is not possible. Further, in addition to the variation in prevalence for patients of different age groups with MDD,^42^ it should be noted that the inability to control for a history of depression may impact this study's findings. In particular, the lower HR reported in males <25 years compared with males ≥25 years (1.00 vs. 1.52) should be interpreted with this in mind. An important additional consideration is the potential for underdiagnosis or a lack of MDD symptom disclosure due to high levels of mental health stigma in Japan, including amongst AYA populations.^43^ Lastly, the number and type of confounders were limited by the availability of data from the anonymized administrative claims.

### 4.2 Clinical implications

There is a need for physicians treating cancer patients to be aware of the high possibility of MDD among AYA patients. As MDD among AYA cancer patients is treatable, it is crucial to look out for depressive symptoms, especially in patients whose cancer has a poor prognosis or are receiving chemotherapy during hospitalization, and ensure that these patients receive appropriate support and treatment. In consideration of the high risk in AYA patients, and their limited access to and use of dedicated mental health resources, a special support system potentially including depression screening, multidisciplinary care, or increased specialization in managing AYA patients may be desirable in Japan.

## 5 CONCLUSIONS

In Japan, AYA patients with cancer had an increased risk for MDD compared with cancer‐free controls. At highest risk were those with multiple cancer categories, brain/CNS cancer, leukemia, and stomach cancer. These patients and those who received aggressive treatment should be monitored closely and provided appropriate support and mental health care.

## CONFLICT OF INTEREST

Tatsuo Akechi is a speaker for Astellas, AstraZeneca, Daiichi Sankyo, Dainippon‐Sumitomo, Eisai, Hisamitsu, Kyowa‐Hakko Kirin, Kyowa, Eli Lilly, MSD, Meiji‐Seika Pharma, Mochida, Mundipharma, Otsuka, Pfizer, Shionogi, and Tsumura; has received grants from Daiichi‐Sankyo, Eisai, FUJIFILM RI Pharma, Eli Lilly, MSD, Novartis, Otsuka, Shionogi, Tanabe‐Mitsubishi, and Takeda, outside the submitted work. Izumi Mishiro and Shinji Fujimoto report personal fees from Takeda during the conduct of the study and outside the submitted work.

## Supporting information

Supplementary Material S1

## Data Availability

The data that support the findings of this study are available from JMDC Inc. but were used under license for the current study; therefore, restrictions apply and the data are not publicly available. For inquiries about access to the data set used in this study, please contact JMDC (https://www.jmdc.co.jp).
