# Supplementary material for: Risk of major depressive disorder in adolescent and young adult cancer patients in Japan
Source: Psychooncology. 2022 Jan 20;31(6):929–37. doi: 10.1002/pon.5881 (PMC9305902; doi:10.1002/pon.5881)
Supplement: Supplementary file 1 — Supplementary Material S1 [file PON-31-929-s001.docx]

# Supporting Information

## Table S1. ICD-10 Codes Used to Classify Cancer Categories

| **Category** | **ICD-10 code** | **ICD-10 term** |  |
| --- | --- | --- | --- |
| Lip, oral cavity, and pharynx | C00 | Malignant neoplasm of lip |  |
|  | C01 | Malignant neoplasm of base of tongue |  |
|  | C02 | Malignant neoplasm of other and unspecified parts of tongue |  |
|  | C03 | Malignant neoplasm of gum |  |
|  | C04 | Malignant neoplasm of floor of mouth |  |
|  | C05 | Malignant neoplasm of palate |  |
|  | C06 | Malignant neoplasm of other and unspecified parts of mouth |  |
|  | C07 | Malignant neoplasm of parotid gland |  |
|  | C08 | Malignant neoplasm of other and unspecified major salivary glands |  |
|  | C09 | Malignant neoplasm of tonsil |  |
|  | C10 | Malignant neoplasm of oropharynx |  |
|  | C11 | Malignant neoplasm of nasopharynx |  |
|  | C12 | Malignant neoplasm of piriform sinus |  |
|  | C13 | Malignant neoplasm of hypopharynx |  |
|  | C14 | Malignant neoplasm of other and ill-defined sites in the lip, oral cavity, and pharynx |  |
| Esophagus | C15 | Malignant neoplasm of esophagus |  |
| Stomach | C16 | Malignant neoplasm of stomach |  |
| Small intestine | C17 | Malignant neoplasm of small intestine |  |
| Colorectum |  | Colon or rectum |  |
| Colon | C18 | Malignant neoplasm of colon |  |
| Rectum | C19 | Malignant neoplasm of rectosigmoid junction |  |
|  | C20 | Malignant neoplasm of rectum |  |
|  | C21 | Malignant neoplasm of anus and anal canal |  |
| Liver | C22 | Malignant neoplasm of liver and intrahepatic bile ducts |  |
| Gallbladder/biliary tract | C23 | Malignant neoplasm of gallbladder |  |
|  | C24 | Malignant neoplasm of other and unspecified parts of biliary tract |  |
| Pancreas | C25 | Malignant neoplasm of pancreas |  |
| Other digestive organs | C26 | Malignant neoplasm of other and ill-defined digestive organs |  |
| Nasal cavity/sinus and middle ear | C30 | Malignant neoplasm of nasal cavity and middle ear |  |
|  | C31 | Malignant neoplasm of accessory sinuses |  |
| Larynx | C32 | Malignant neoplasm of larynx |  |
| Lung | C33 | Malignant neoplasm of trachea |  |
|  | C34 | Malignant neoplasm of bronchus and lung |  |
| Other intrathoracic organs | C37 | Malignant neoplasm of thymus |  |
|  | C38 | Malignant neoplasm of heart, mediastinum, and pleura |  |
| Bone and articular cartilage | C40 | Malignant neoplasm of bone and articular cartilage of limbs |  |
|  | C41 | Malignant neoplasm of bone and articular cartilage of other and unspecified sites |  |
| Skin | C43 | Malignant melanoma of skin |  |
|  | C44 | Other malignant neoplasms of skin |  |
| Mesothelium and soft tissue | C45 | Mesothelioma |  |
|  | C46 | Kaposi's sarcoma |  |
|  | C47 | Malignant neoplasm of peripheral nerves and autonomic nervous system |  |
|  | C48 | Malignant neoplasm of retroperitoneum and peritoneum |  |
|  | C49 | Malignant neoplasm of other connective and soft tissue |  |
| Breast | C50 | Malignant neoplasm of breast |  |
| Uterine cervix | C53 | Malignant neoplasm of cervix uteri |  |
| Uterine corpus | C54 | Malignant neoplasm of corpus uteri |  |
| Ovary | C56 | Malignant neoplasm of ovary |  |
| Other female genitalia | C51 | Malignant neoplasm of vulva |  |
|  | C52 | Malignant neoplasm of vagina |  |
|  | C57 | Malignant neoplasm of other and unspecified female genital organs |  |
|  | C58 | Malignant neoplasm of placenta |  |
| Prostate | C61 | Malignant neoplasm of prostate |  |
| Other male genitalia | C60 | Malignant neoplasm of penis |  |
|  | C62 | Malignant neoplasm of testis |  |
|  | C63 | Malignant neoplasm of other and unspecified male genital organs |  |
| Bladder | C67 | Malignant neoplasm of bladder |  |
| Kidney/urinary tract (excl. bladder) | C64 | Malignant neoplasm of kidney, except renal pelvis |  |
|  | C65 | Malignant neoplasm of renal pelvis |  |
|  | C66 | Malignant neoplasm of ureter |  |
|  | C68 | Malignant neoplasm of other and unspecified urinary organs |  |
| Eye | C69 | Malignant neoplasm of eye and adnexa |  |
| Brain/central nervous system | C70 | Malignant neoplasm of meninges |  |
|  | C71 | Malignant neoplasm of brain |  |
|  | C72 | Malignant neoplasm of spinal cord, cranial nerves, and other parts of central nervous system |  |
| Thyroid gland | C73 | Malignant neoplasm of thyroid gland |  |
| Other endocrine glands | C74 | Malignant neoplasm of adrenal gland |  |
|  | C75 | Malignant neoplasm of other endocrine glands and related structures |  |
| Other malignant neoplasms | C76 | Malignant neoplasm of other and ill-defined sites |  |
|  | C77 | Secondary and unspecified malignant neoplasm of lymph nodes |  |
|  | C78 | Secondary malignant neoplasm of respiratory and digestive organs |  |
|  | C79 | Secondary malignant neoplasm of other and unspecified sites |  |
|  | C80 | Malignant neoplasm, without specification of site |  |
| Malignant lymphoma | C81 | Hodgkin lymphoma |  |
|  | C82 | Follicular lymphoma |  |
|  | C83 | Non-follicular lymphoma |  |
|  | C84 | Mature T/NK-cell lymphomas |  |
|  | C85 | Other and unspecified types of non-Hodgkin lymphoma |  |
|  | C86 | Other specified types of T/NK-cell lymphoma |  |
|  | C96 | Other and unspecified malignant neoplasms of lymphoid, hematopoietic, and related tissue |  |
| Multiple myeloma | C88 | Malignant immunoproliferative diseases |  |
|  | C90 | Multiple myeloma and malignant plasma cell neoplasms |  |
| Leukemia | C91 | Lymphoid leukemia |  |
|  | C92 | Myeloid leukemia |  |
|  | C93 | Monocytic leukemia |  |
|  | C94 | Other leukemias of specified cell type |  |
|  | C95 | Leukemia of unspecified cell type |  |
| Multiple categories | ≥2 of the above categories | | |

Abbreviations: excl., excluding; ICD-10, International Statistical Classification of Diseases and Related Health Problems, 10th revision.^10^ NK, natural killer.

## Table S2. Multivariate Analyses of Time-to-MDD with AYA Patients with Cancer and AYA Controls, by Cancer Category

| **Cancer Category^†^ Variable** | **Reference** | **Category** | **Hazard Ratio^§^ (95% CI)** |
| --- | --- | --- | --- |
| *Oral cavity, pharynx* | | | |
| Group | Control group | AYA patients with cancer | 1.99 (0.44-9.06) |
| Sex | Male | Female | 0.82 (0.21-3.28) |
| Working status | Working | Non-working | 0.27 (0.03-2.38) |
| *Esophagus* |  |  |  |
| Group | Control group | AYA patients with cancer | 4.91 (0.44-54.11) |
| Sex | Male | Female | 2.99 (0.27-32.97) |
| Working status | Working | Non-working | — |
| *Stomach* |  |  |  |
| Group | Control group | AYA patients with cancer | 5.80 (2.43-13.83) |
| Sex | Male | Female | 1.41 (0.44-4.51) |
| Working status | Working | Non-working | 0.78 (0.22-2.82) |
| *Small intestine* | | | |
| Group | Control group | AYA patients with cancer | NE |
| Sex | Male | Female | 1.77 (0.15-20.55) |
| Working status | Working | Non-working | 0.88 (0.08-10.27) |
| *Colorectum* |  |  |  |
| Group | Control group | AYA patients with cancer | 3.26 (1.82-5.83) |
| Sex | Male | Female | 1.17 (0.63-2.16) |
| Working status | Working | Non-working | 0.73 (0.35-1.54) |
| *Liver* |  |  |  |
| Group | Control group | AYA patients with cancer | 2.03 (0.44-9.26) |
| Sex | Male | Female | 1.45 (0.36-5.75) |
| Working status | Working | Non-working | 0.40 (0.08-1.92) |
| *Gallbladder, bile duct* | | | |
| Group | Control group | AYA patients with cancer | NE |
| Sex | Male | Female | 7.47 (0.68-82.46) |
| Working status | Working | Non-working | 0.16 (0.01-1.72) |
| *Pancreas* |  |  |  |
| Group | Control group | AYA patients with cancer | 3.43 (0.69-17.00) |
| Sex | Male | Female | NE |
| Working status | Working | Non-working | NE |
| *Lung* |  |  |  |
| Group | Control group | AYA patients with cancer | 3.84 (1.50-9.80) |
| Sex | Male | Female | 0.65 (0.21-1.99) |
| Working status | Working | Non-working | 1.41 (0.45-4.47) |
| *Other intrathoracic organ* | | | |
| Group | Control group | AYA patients with cancer | 10.00 (0.63-159.88) |
| Sex | Male | Female | NE |
| Working status | Working | Non-working | 12.00 (0.75-191.85) |
| *Skin* |  |  |  |
| Group | Control group | AYA patients with cancer | 2.72 (0.90-8.21) |
| Sex | Male | Female | 0.48 (0.17-1.36) |
| Working status | Working | Non-working | 1.85 (0.67-5.10) |
| *Mesothelium and soft tissue* | | | |
| Group | Control group | AYA patients with cancer | 1.83 (0.40-8.24) |
| Sex | Male | Female | 1.19 (0.36-3.94) |
| Working status | Working | Non-working | 0.39 (0.08-1.95) |
| *Breast^a^* |  |  |  |
| Group | Control group | AYA patients with cancer | 3.88 (2.49-6.05) |
| Sex | Female | Male | 2.86 (0.40-20.65) |
| Working status | Working | Non-working | 0.98 (0.66-1.47) |
| *Uterine cervix^‡^* | | | |
| Group | Control group | AYA patients with cancer | 1.37 (0.58-3.20) |
| Sex | Female | Male | NE |
| Working status | Working | Non-working | 0.87 (0.50-1.52) |
| *Uterine corpus^a^* | | | |
| Group | Control group | AYA patients with cancer | NE |
| Sex | Female | Male | 1.26 (0.75-2.10) |
| Working status | Working | Non-working | 0.45 (0.17-1.18) |
| *Ovary^a^* |  |  |  |
| Group | Control group | AYA patients with cancer | 3.89 (1.94-7.79) |
| Sex | Female | Male | NE |
| Working status | Working | Non-working | 0.82 (0.44-1.52) |
| *Prostate gland* | | | |
| Group | Control group | AYA patients with cancer | NE |
| Sex | Male | Female | - |
| Working status | Working | Non-working | - |
| *Other male genitalia* | | | |
| Group | Control group | AYA patients with cancer | 2.53 (1.03-6.18) |
| Sex | Male | Female | - |
| Working status | Working | Non-working | NE |
| *Bladder* |  |  |  |
| Group | Control group | AYA patients with cancer | 1.00 (0.13-7.78) |
| Sex | Male | Female | 2.92 (0.68-12.43) |
| Working status | Working | Non-working | 0.13 (0.01-1.19) |
| *Kidney, urinary tract (except bladder)* | | | |
| Group | Control group | AYA patients with cancer | 1.85 (0.41-8.36) |
| Sex | Male | Female | 0.78 (0.14-4.25) |
| Working status | Working | Non-working | 0.50 (0.05-4.95) |
| *Brain, central nervous system* | | | |
| Group | Control group | AYA patients with cancer | 4.13 (1.82-9.39) |
| Sex | Male | Female | 1.91 (0.81-4.51) |
| Working status | Working | Non-working | 0.48 (0.20-1.19) |
| *Thyroid gland* |  |  |  |
| Group | Control group | AYA patients with cancer | 1.32 (0.60-2.91) |
| Sex | Male | Female | 0.91 (0.48-1.74) |
| Working status | Working | Non-working | 1.16 (0.64-2.10) |
| *Other malignant neoplasm* | | | |
| Group | Control group | AYA patients with cancer | 2.71 (1.01-7.25) |
| Sex | Male | Female | 2.92 (1.11-7.71) |
| Working status | Working | Non-working | 0.82 (0.36-1.91) |
| *Malignant lymphoma* | | | |
| Group | Control group | AYA patients with cancer | 2.94 (1.66-5.22) |
| Sex | Male | Female | 0.89 (0.51-1.55) |
| Working status | Working | Non-working | 0.60 (0.33-1.10) |
| *Multiple myeloma* | | | |
| Group | Control group | AYA patients with cancer | 1.25 (0.16-9.96) |
| Sex | Male | Female | 0.95 (0.22-4.15) |
| Working status | Working | Non-working | 0.68 (0.16-2.96) |
| *Leukemia* |  |  |  |
| Group | Control group | AYA patients with cancer | 6.30 (3.75-10.58) |
| Sex | Male | Female | 0.92 (0.51-1.64) |
| Working status | Working | Non-working | 1.33 (0.74-2.37) |
| *Multiple categories* | | | |
| Group | Control group | AYA patients with cancer | 6.73 (3.65-12.40) |
| Sex | Male | Female | 0.73 (0.35-1.51) |
| Working status | Working | Non-working | 1.27 (0.60-2.68) |

^†^Cancer categories with >100 patients.

^‡^Includes data only from female patients.

^§^Adjusted for sex and working status.

Abbreviations: AYA, adolescent and young adult; CI, confidence interval; MDD, major depressive disorder; NE, not evaluable.

## Table S3. Multivariate Analysis of HRs of MDD Incidence and 95% CI Between Subgroups of AYA Patients with Cancer

| Variable | Reference | Category | Hazard Ratio^†^ (95% CI) |
| --- | --- | --- | --- |
| Sex | Male | Female | 1.08 (0.76-1.04) |
| Sex, age (years) | Male, <25 | Male, ≥25 | 0.64 (0.35-1.17) |
|  |  | Female, <25 | 0.70 (0.33-1.50) |
|  |  | Female, ≥25 | 0.82 (0.49-1.50) |
| Working status | Working | Non-working | 1.23 (0.88-1.76) |
| Chemotherapy | Inpatient | None | 0.43 (0.30-0.62) |
|  |  | Outpatient only | 0.75 (0.41-1.37) |
| Radiation therapy | External irradiation | None | 1.35 (0.58-3.12) |
|  |  | Brachytherapy only | - |
| Surgery with over 5 days hospitalization | Yes | No | 0.96 (0.70-1.33) |

^†^Adjusted for all covariates shown.

Abbreviations: AYA, adolescent and young adult; CI, confidence interval; MDD, major depressive disorder.

## Fig.S1. Study Design


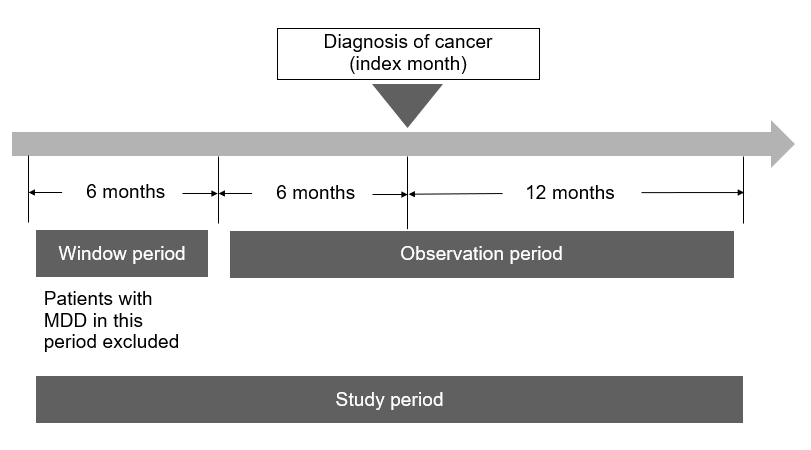


Abbreviation: MDD, major depressive disorder

## Fig.S2. Flow Chart for Selection of AYA Patients with Cancer and AYA Cancer-Free Controls Included in the Study Population

Abbreviations: AYA, adolescent and young adult; MDD, major depressive disorder.
